# ICTD: Combination of Improved CNN–Transformer and Enhanced Deep Canonical Correlation Analysis for Eye-Movement Emotion Classification

**DOI:** 10.3390/brainsci16030330

**Published:** 2026-03-19

**Authors:** Cong Zhang, Xisheng Li, Jiannan Chi, Ming Cao, Qingfeng Gu, Jiahui Liu

**Affiliations:** 1 School of Automation and Electrical Engineering, University of Science and Technology Beijing, Beijing 100083, Chinaustbjnc@ustb.edu.cn (J.C.);; 2 Shunde Innovation School, University of Science and Technology Beijing, Foshan 528399, China

**Keywords:** eye-movement, emotion classification, ICTD, incremental feature feedforward network, CNN-transformer

## Abstract

**Highlights:**

**What are the main findings?**

- This paper proposes a deep canonical correlation analysis method based on cosine similarity, non-linearly transforming feature vectors of different modalities into feature vectors with stronger correlation to improve the accuracy of emotion classification.
- This paper proposes an incremental feature feedforward network (IFFN) to perform feature transformations in enhancement and simplification, replacing the FFN in the original transformer module.

**What are the implications of the main findings?**

- Cosine similarity pays more attention to the direction of vectors than it does to their magnitude, is affected less by outliers, and does not require the data to satisfy specific distribution assumptions. The characteristics that are more suitable for eye-movement input data have provided a suitable processing method for eye-movement-based emotion classification.
- Existing studies mostly rely on the statistical characteristics of the original data for emotion analysis. Moreover, the importance of each emotional feature in the calculation process is not primary or secondary, and the role of key features cannot be highlighted. By assigning higher weights to key features, the influence of indiscriminately input features is reduced, thereby enhancing the importance of key features. This design effectively addresses the lack of prioritization in feature importance and significantly improves the ability of eye-movement features to characterize emotional states.

**Abstract:**

**Background/Objectives**: Emotion classification based on eye-movement features has become a widely adopted approach due to the simplicity of data acquisition and the strong association between ocular responses and emotional states. However, several challenges remain with regard to existing emotion recognition methods, including the relatively weak correlation between eye-movement features and emotional labels and the fact that the key features are not prominently presented. **Methods:** To address abovelimitations, this study proposes an improved CNN-transformer combined with enhanced deep canonical correlation analysis network (ICTD). The proposed method first performs preprocessing and reconstruction of raw eye-movement signals to extract informative features. Subsequently, convolutional neural networks (CNNs) and transformer architectures are employed to capture local and global feature, respectively. In addition, an incremental feature feedforward network is incorporated to enhance the transformer, enabling the model to assign higher importance to salient feature information. Finally, the extracted representations are processed through deep canonical correlation analysis based on cosine similarity in order to generate classification outcomes. **Results:** Experiments conducted on the SEED-IV, SEED-V, and eSEE-d datasets demonstrate that the proposed ICTD framework consistently outperforms baseline approaches and attains optimal classification results. (1) On the eSEE-d dataset, the results of three-category arousal and valence classification reach 81.8% and 85.2%, respectively; (2) on the SEED-IV dataset, the emotion four-category classification result reaches 91.2%; (3) finally, on the SEED-V dataset, the emotion five-category classification result reaches 85.1%. **Conclusions:** The proposed ICTD framework effectively improves feature representation and classification performance, showing strong potential for practical emotion recognition and physiological signal analysis.

## 1. Introduction

Emotion is a subjective physiological phenomenon of humans, and the expression of emotion is multifaceted. Accurately identifying emotional states plays a crucial role in cognitive science [1]. Picard [2] proposed the concept of affective computing, aiming to research and develop systems and devices capable of recognizing, interpreting, processing, and simulating human emotions [3]. Emotion recognition in humans has become an important area of research within affective computing, significantly contributing to both the management of emotional states and the identification of disorders linked to emotions [4].

In recent years, researchers have conducted a series of studies on the acquisition and classification of emotional states [5,6,7,8]. Currently, methods for acquiring emotional data are categorized into non-contact- and contact-based techniques. Contact methods typically involve physiological sensors such as electroencephalography (EEG), electrocardiography (ECG), and electrodermal activity (EDA). It is worth mentioning the functional near-infrared spectroscopy (fNIRS) technique for emotion detection, which has grown in popularity in recent years. Their main characteristics are the need to fix devices on the subject and the high requirements for the acquisition environment [9,10,11]. Non-contact methods primarily involve the analysis of voice, facial expressions, and eye-tracking signals. These approaches offer the benefits of straightforward signal collection while ensuring that participants experience no discomfort [12,13]. The study of eye-tracking signals has become an ideal research subject in affective computing due to the ease of their acquisition and their direct correlation with emotions. Acquiring eye-tracking signals does not require wearing cumbersome devices like EEG or ECG signals. Eye-tracking data primarily include fixation, saccade, and pupil-related information. The frequency and duration of fixations provide direct insights into the subject’s level of attention and focus on specific stimuli. Saccades, being the most frequent type of eye-movement, exhibit variations in speed and duration, which reflect different levels of attention directed toward distinct targets. Changes in pupil size are positively correlated with the current stimulus experienced by the subject. When individuals are in different emotional states, such as happiness, sadness or fear, their eye-tracking behavior shows systematic differences that are specifically reflected in fixation duration, saccade speed, and pupil diameter, among other aspects. For example, individuals in a happy state often exhibit longer fixation times on positive stimulus areas, while, in a fearful state, their pupils tend to dilate significantly, something which is accompanied by high-frequency saccadic behavior [14]. The combined influence of eye-movement characteristics and the inherent relationships between emotions and these features demonstrates that emotion recognition based on eye-tracking is both feasible and scientifically grounded. In recent years, a range of deep neural networks has been applied to affective computing, and their outstanding performance indicates that they surpass traditional shallow approaches in effectiveness [15]. Although eye-tracking features demonstrate numerous advantages in affective computing, there are still some major issues that limit the improvement of emotional computing accuracy.

This study proposes an improved CNN–transformer framework integrated with an enhanced deep canonical correlation analysis network for emotion recognition. The proposed approach comprehensively incorporates multiple eye-movement features into the emotion classification process, enabling more effective feature extraction and classification. These features provide richer representations of emotional characteristics and contribute to improved discrimination among different emotional states. The CNN–transformer architecture is employed to capture both local and global feature information. In addition, the conventional transformer structure is enhanced by introducing an incremental feature feedforward network, which allows important feature information to receive greater attention during feature learning. Finally, deep canonical correlation analysis based on cosine similarity is applied to the extracted features, and the final classification results are obtained by measuring the correlations between the learned feature representations.

The remaining part of this paper is structured as follows. Section 2 provides a summary of the common methods and network models for eye-movement emotion analysis. Section 3 details the overall structure of the ICTD algorithm and the implementation methods of each module. Section 4 introduces the dataset employed in the experiments, the experimental results, and the comparison results with baseline methods. Section 5 discusses the contributions of ICTD as well as its existing shortcomings. Finally, Section 6 introduces the conclusions and future work.

## 2. Related Work

Eye-tracking technology has been widely applied across numerous domains, including applied psychology, neuropsychology, and medical research [16,17,18,19], and has become an increasingly prominent analytical tool in recent years. Existing studies on emotion recognition primarily focus on improving classification performance through multimodal data integration [20], while comparatively limited attention has been devoted to exploring the intrinsic information embedded in eye-tracking signals themselves. As a result, although eye-tracking has been extensively utilized as a supporting modality in emotion analysis, research relying solely on eye-tracking parameters for emotion recognition remains relatively limited and is still an emerging methodological direction. Therefore, this section aims to systematically review the current research progress and identify the key challenges associated with emotion analysis based exclusively on eye-tracking parameters.

### 2.1. Eye-Movement Features for Emotion Recognition

Given the widespread interest in emotion recognition tasks using eye-tracking features, an increasing number of methods for assessing emotional states have been proposed. In the research on the affective analysis of eye-tracking features, Oliva et al. [21] investigated how variations in pupil diameter relate to emotion recognition using nonverbal vocal stimuli, and their findings indicated that pupil size changes are associated with cognitive processing [22]. Changes in baseline pupil size are related to task efficiency. The pupil diameter increases during task interruptions, while it decreases during task engagement. The study aimed to test stimuli with varying values, intensity, duration, and recognition difficulty. Aracena et al. [23] randomly selected 90 images from the IAPS dataset and categorized them into three emotional classes: positive, neutral, and negative, while also evaluating them according to three corresponding valence levels. Classification was subsequently conducted using neural networks [24] and binary decision trees. The best performance, obtained with DeepLearntools [25], reached a recognition rate of 82.8%, with a mean accuracy of 71.7%. Skaramagkas et al. [26] used eye-tracking features such as fixation duration, saccade amplitude, etc., and selected 48 participants to watch 10 emotion-inducing videos, recording their ocular responses. The authors classified emotions based on statistical features of the eye ocular responses, including fixation and blink frequency, saccade amplitude and duration, pupil diameter, fixation duration kurtosis, saccade duration variation and pupil diameter kurtosis. Participants evaluated four emotional states: tenderness, anger, disgust, and sadness, and a deep multilayer perceptron (DMLP) model achieved 92% accuracy in discriminating positive versus non-positive valence, as well as 84% accuracy in differentiating low from medium arousal.

### 2.2. Ocular Response-Based Emotional Computing for Differentiated Individuals

Emotional data collected from different subjects typically exhibits high individual variability, and current research focuses on model generalization studies to reduce inter-individual differences. Fu et al. [27] proposed a multimodal feature fusion neural network (MFFNN) designed to effectively extract complementary information from eye-movement signals and integrate it with EEG features. Their architecture employs a dual-branch feature extraction module to capture features from both modalities while maintaining temporal alignment. Additionally, a multi-scale feature fusion module is incorporated, which leverages cross-channel soft attention to selectively aggregate information from different spatial scales, thereby enabling effective multi-scale feature integration. Experiments conducted on the publicly available SEED-IV dataset demonstrate that the proposed model achieves an accuracy of 87.32% for four-class emotion recognition, encompassing happiness, sadness, fear, and neutral. Li et al. [28] employed a deep gradient convolutional neural network (DGCNN) by inputting eye-tracking signal images processed with FFT into the network, achieving an 87% accuracy in recognizing four emotional categories: nervous, calm, happy, and sad. Tarnowski et al. [29] utilized 18 eye-movement features to classify emotional states, including high arousal/negative valence, low arousal/medium valence, and high arousal/positive valence. The study employed three classifiers—support vector machines, linear discriminant analysis, and k-nearest neighbors—and evaluated performance using a leave-one-subject-out cross-validation approach. The results indicated a peak classification accuracy of 80%, with the strongest performance observed in distinguishing high and low arousal emotions. Wang et al. [30] collected and analyzed electrooculogram (EOG) signals and eye-tracking videos simultaneously, proposing an emotion perception algorithm based on eye-tracking information. They applied short-time Fourier transform (STFT) to the original multi-channel EOG signals to extract time–frequency eye-tracking features, integrated time–domain eye-tracking features using two feature fusion strategies, and conducted recognition experiments on positive, neutral, and negative emotional states, achieving average respective accuracies of 88.64% and 88.35%.

### 2.3. Multimodal Emotion Analysis Model with Eye-Tracking Data

In recent years, scholars have carried out research on emotion analysis by utilizing multimodal physiological signals including electroencephalogram (EEG) signals, electrocardiogram (ECG) signals, electrooculogram (EOG) signals, skin conductance, and eye-movement signals. Among these, the combination of EEG and eye-movement for emotion analysis is a relatively prevalent approach. Gong et al. [31] put forward a novel multimodal emotion recognition model, named integrity-induced adaptive broad learning (CiABL), to enhance the generalization performance for unseen target domain topics. This introduces a weighted representation distribution alignment mechanism that is capable of effectively aligning both marginal and conditional distributions, thereby mitigating the influence of individual differences. Experiments on the SJTU emotion EEG dataset (SEED) and SEED-FRA datasets verified its effectiveness and generalization ability, and it outperformed the current mainstream methods. Magdiel proposed a multimodal semi-supervised domain adaptation method, namely cross modal joint distribution alignment (CMJDA), which conducts cross modal learning and joint distribution alignment by leveraging the discriminative features of each modality through independent neural networks [32]. The model generates correlated features and consistent predictions between modalities and promotes the similarity of marginal and conditional distributions among labeled source data, limited labeled target data, and a large amount of unlabeled target data. Comparative experiments were conducted via “Leave-One-Subject -Out” cross-validation on two public emotion recognition datasets, SEED-IV and SEED-V. An average accuracy ranging from 92.50% to 96.13% was achieved in three available sessions of SEED-IV and SEED-V. Liu et al. [33] studied how people from China, Germany, and France recognize emotions by analyzing both EEG signals and eye-movement data. A systematic analysis was performed across four dimensions: emotion model performance, neural patterns, complementary information from multiple modalities, and cross-cultural emotion recognition to evaluate emotion recognition accuracy. The study concluded that, in terms of cross-cultural emotion recognition, German and French participants exhibit greater similarity in terrain patterns and attention weight distributions when compared with Chinese participants. Additionally, while Chinese data align well with test datasets, it is less suitable for training models for the other two cultural groups.

Based on the above research, the research methods and characteristics of different ocular response-based emotion classification studies are summarized as shown in Table 1.

From the above studies, several challenges remain in the field of eye-movement-based emotion classification.

(1) The correlation between eye-movement features and emotional states remains relatively weak. Emotional responses are complex and cannot be accurately represented by only a limited number of eye-movement features. In practice, the same eye-movement feature may exhibit different patterns across different emotional states [34]. Therefore, further investigation is required to better understand the relationships between emotional states and eye-movement features, as well as to enhance and identify discriminative feature representations to improve classification performance.

(2) Eye-movement features are often treated merely as auxiliary information in multimodal emotion recognition frameworks, which limits their independent analytical potential. For example, Zheng [35] combined EEG features and eye-movement features through a linear fusion strategy to classify four emotional states, achieving an average accuracy of 72%, whereas the accuracy obtained using only eye-movement features was 67%. This indicates that studies focusing solely on eye-movement-based emotion classification remain limited, and that methodological developments specifically designed for eye-movement signals are still insufficient.

(3) Most existing studies rely on statistical feature extraction from eye-movement data for emotion analysis, without differentiating the relative importance of individual features during the computational process. As a result, the contribution of key features cannot be sufficiently emphasized. When a large number of features are included as input, the weights of features with strong emotional representation capability may be diluted, thereby negatively affecting the overall emotion classification performance.

## 3. Proposed Method

### 3.1. Task Definition

In this research, the data samples in the dataset are represented as $\left\{X\in R^{N\times D}\right\}$, where N indicates the total number of samples and D denotes the feature dimensionality. The target set Y is defined as $Y=\left\{Y_{i}\right\},Y_{i}\in \left\{0,1,\ldots ,C\right\}$, which corresponds to the set of emotion categories, with *C* representing the number of classes. The primary objective of this work is to extract features from X and predict the corresponding emotion category Y.

### 3.2. The Structure of the Proposed Method

**Figure 1:**
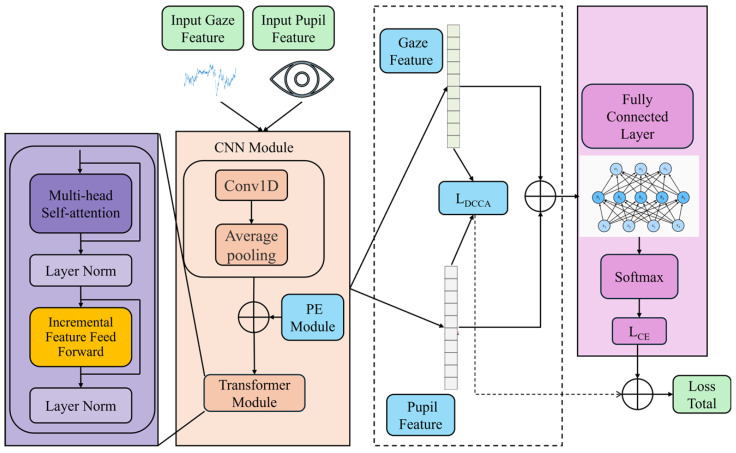
Structure diagram of ICTD.

This paper presents an improved CNN–transformer combined with enhanced deep canonical correlation analysis module based on cosine similarity, as depicted in Figure 1.

In this framework, pupil diameter data and gaze data extracted from eye-tracking signals are treated as two independent yet correlated modalities. These modalities are first processed through separate CNN branches for hierarchical feature extraction, enabling the capture of local feature details. The extracted CNN features are subsequently fed into a transformer encoder. Within the transformer architecture, the conventional feedforward network (FFN) is replaced with an incremental feature feedforward network (IFFN), which is designed to enhance the weighting of salient features. Through the self-attention mechanism, the transformer models the internal dependencies among features and generates context-enhanced feature representations. The resulting feature representations from the two modalities are then input into a deep canonical correlation analysis (DCCA) module. Through projection networks, the features are mapped into a shared latent space and optimized using canonical correlation analysis to maximize the cosine similarity between the projected representations of the two modalities. Finally, the aligned dual-modal features are fused and fed into a classifier. By jointly optimizing classification loss and correlation loss, the proposed framework simultaneously enhances feature discriminability and inter-modal consistency, thereby achieving accurate multimodal emotion classification. The details of each component in the proposed architecture are described as follows.

#### 3.2.1. CNN–Transformer Module

As the videos watched by the participants contain strong emotional incentives, this leads to sudden changes in the participants’ emotions, and the emotions generated gradually disappear as the video changes. Therefore, the data during the period from the generation to the disappearance of emotional incentives form an important feature in the emotional computing process and a decisive factor in ensuring the accuracy of emotional computing. In the experiments of this paper, the eye-tracking features used include both local features such as pupil size and fixation duration, as well as global statistical features such as saccade time kurtosis and fixation frequency. Thus, the model needs to accurately process local features while also having strong processing capabilities for global features.

The transformer architecture primarily relies on self-attention mechanisms to handle dependencies in the input sequence, exhibiting excellent performance in long sequence modeling. In contrast, CNN excel at handling local features and weight sharing, making them well suited for extracting local features. Therefore, embedding CNN into transformer models aims to combine the ability of CNN to capture local features with the advantages of transformers in modeling long-range dependencies, thereby improving the overall feature representation capability and prediction performance of the model.

To effectively model temporal dynamics at different resolutions, multiple average pooling layers with kernel sizes of 1 × 8, 1 × 16, 1 × 32, 1 × 64, 1 × 128, 1 × 256, and 1 × 512 are introduced in the CNN module. These pooling operations perform temporal aggregation over windows of different lengths, enabling the extraction of multi-scale temporal features. Specifically, smaller pooling kernels capture fine-grained short-term variations, while larger kernels encode long-range temporal dependencies and global trends. The integration of multi-scale pooled features provides richer temporal representations, which benefits subsequent sequence modeling in the transformer module.

Based on the characteristics of the aforementioned model, this study used two CNN-based modules to process the transformer encoder and decoder. The two CNN modules are employed to extract local features from the input data via one-dimensional convolutional layers. Within the encoder, a two-layer convolutional structure was implemented to increase the output feature channels to 128. The first convolutional layer utilizes a kernel size of 3, with 5 input channels and 64 output channels; the second layer also uses a kernel size of 3 and produces 128 output channels. Each convolution is followed by a ReLU activation, and the same padding is applied to preserve the time-step length, thereby maintaining the integrity of the temporal features. To prevent data redundancy from causing model overfitting, we set the number of heads of the transformer to 8 and the number of layers to 2.

Positional encoding plays a crucial role in transformer models, as the self-attention mechanism itself cannot sense the order of input sequences. To compensate for this shortcoming, position coding enables the model to obtain position information about the individual elements in the sequence, thereby helping to understand the sequential relationships of the input data. By combining element content and position information, position coding not only enhances the model’s expressive ability but also effectively avoids the common gradient disappearance problem in RNNs, making the transformer perform better when dealing with long-distance dependencies [36]. Position coding is typically calculated using fixed sine and cosine functions, whose values are closely related to position information in the sequence. For a given sequence position POS and encoding dimensions, the formula for calculating position coding is as follows:

(1) $$PE_{\left(pos,2i\right)}=\sin\left(\frac{pos}{{10,000}^{\frac{2i}{d}}}\right)$$

(2) $$PE_{\left(pos,2i+1\right)}=\cos\left(\frac{pos}{{10,000}^{\frac{2i}{d}}}\right)$$

where pos is the position index of the element in the sequence, i is the dimension index, and d is the dimension of the input vector. This sine and cosine encoding method has smooth variation characteristics and can provide the model with relative position information between different positions.

In the architecture of the proposed model, an improved multi-layer transformer encoder module is used as the sequence model. The encoder module has two sub-layers: a multi-head self-attention layer as the first part, followed by a positionally fully connected feedforward network. Additionally, each encoder includes residual connections and layer normalization. For supervised classification, a softmax layer is added to the overall network to complete the classification task. During classification, let the labeled dataset be S, which consists of different eye-tracking feature vectors, namely $S=[S_{1},\;\ldots ,S_{n}]$. The input sequence is passed through a feature extraction layer and a multi-layer transformer encoder module to obtain the activation $m_{l}$ of the last encoder. Then, a feedforward layer with parameter $W_{y}$ and a SoftMax layer are added to predict y.

(3) $$P\left(y|S_{1},\ldots ,S_{n}\right)=\mathrm{softmax}\left(m_{l}W_{y}\right)$$

In Equation (3), the conditional probability *P* is modeled using a neural network. Subsequent models will be described below.

#### 3.2.2. Incremental Feature Feedforward Network

In classical transformer architectures, the standard feedforward feature network independently processes each input feature, significantly enhancing the representation of features by leveraging the self-attention mechanism. However, traditional models do not prioritize emotion features during computation, failing to highlight the importance of key features. Therefore, this paper proposes an incremental feature feedforward network (IFFN) to perform feature transformations in enhancement and simplification, replacing the FFN in the original transformer module. The IFFN is constructed by introducing partial convolution (PConv) operations to enhance the information elements in the features. Enhancing existing features strengthens the weights of key features, thereby improving the overall model accuracy. The specific process is shown in Algorithm 1.
**Algorithm 1:** Incremental Feature Feedforward Network.Input:  $\hat{X}$: Autoencoder encoding-decoding and reconstructing the input features Output:   ${\hat{X}}_{out}$: Enhanced output features  Step 1: Partial Convolution Projection    $\left(\hat{X})\leftarrow \mathrm{GELU}(W_{1}\cdot \mathrm{PConv}(\hat{X})\right)$  Step 2: Channel Splitting    $\left({\hat{X}}_{1},{\hat{X}}_{2})\leftarrow \mathrm{Split}(\hat{X}\right)$  Step 3: Cross-Channel Interaction    $\left({\hat{X}}_{r})\leftarrow {\hat{X}}_{1}⊗Conv({\hat{X}}_{2}\right)$  Step 4: Final Projection    $\left({\hat{X}}_{out})\leftarrow \mathrm{GELU}(W_{2}\cdot {\hat{X}}_{r}\right)$  Step 5: Return Result    Return ${\hat{X}}_{out}$

W_1_ and W_2_ represent linear projection, the dim of W_1_ and W_2_ is 128 × 128. PConv(·) refers to the partial convolution [37] operation. Partial convolution introduces a mask on the basis of traditional convolution, and only performs convolution operation on the effective region of the input features. It can ignore missing or invalid regions, thereby realizing the “emphasis” operation of key features. The process is represented by splitting the input $\hat{X}$ after encoding and decoding by the autoencoder, dividing the input into two parts. The purpose is to embed local spatial information into the features [38]. The features of the first branch ${\hat{X}}_{1}$ are multiplied element-wise with the features of the second branch ${\hat{X}}_{2}$ after “localization processing”, so that the local spatial importance weight extracted by the second branch can be added to the features of the first branch.

The purpose of this operation is to attach the key features of the second branch to the features of the first branch. By adjusting the features of the first branch with a dynamic, content-related spatial weight, the weight of the important features is increased, thereby improving the accuracy of the overall model.

#### 3.2.3. Deep Canonical Correlation Analysis Model Based on Cosine Similarity

Deep learning-related analysis methods can learn two nonlinear projections with the help of deep neural networks, non-linearly transforming feature vectors of different modalities into feature vectors with stronger correlation [39]. The optimization objective is to maximize the total correlation coefficient, and its principle can be described as follows:

Suppose the features in the original input deep neural network are $H_{1}\in {\mathbb{R}}^{n\times d_{1}}$ and $H_{2}\in {\mathbb{R}}^{n\times d_{2}}$, where n is the number of samples $d_{1}$ and $d_{2}$ are the input dimensions of the features. The corresponding nonlinear transformations of the deep neural networks are $f_{1}(X_{1};\alpha_{1})$ and $f_{2}(X_{2};\alpha_{2})$, which are the parameters of the two deep neural networks. Let $H_{1}^{\prime }\in {\mathbb{R}}^{o\times n}$ and $H_{2}^{\prime }\in {\mathbb{R}}^{o\times n}$ be the new features obtained after undergoing the nonlinear transformations of their respective deep neural networks, where o is the output dimension, and n is the number of input data.

The goal of deep canonical correlation analysis (DCCA) is to jointly learn the parameters $\alpha_{1}$ and $\alpha_{2}$ of two neural networks so that the cosine similarity correlation between $H_{1}^{\prime }$ and $H_{2}^{\prime }$ is as high as possible, namely:

(4) $$\left(\alpha_{1},\alpha_{2}\right)=\arg\;\max\;\mathrm{Corr}\left(f_{1}\left(H_{1}^{\prime };\alpha_{1}\right),f_{2}\left(H_{2}^{\prime };\alpha_{2}\right)\right)$$

In the process of calculating the correlation coefficient in DCCA, the feature vectors need to be centralized. This centralization operation may cause the data to lose their original distribution characteristics, thereby introducing bias. Additionally, in the presence of outliers or extreme values, the centralization effect may be poor, and gradient explosion may occur when using maximum correlation coefficient as a constraint during experiments. To address these issues, an alternative correlation measurement method is adopted: cosine similarity. Using cosine similarity allows one to largely avoid the problems encountered by the correlation coefficient and it has the following advantages when compared with the correlation coefficient:

(1) Cosine similarity pays more attention to the direction of vectors rather than their magnitude, making it more suitable for measuring directional similarity between two feature vectors. During the process of subjects watching videos, the eye-tracking features and gaze features may exhibit similar structural changes in their degree of variation, and the similarity in spatial direction of this information can be better reflected.

(2) Cosine similarity does not require the data to follow specific distributional assumptions. In practical scenarios, the data may be influenced by complex environments and varying objects, which can lead to deviations from strict Gaussian distributions. Therefore, cosine similarity is more suitable for measuring the similarity of such data.

(3) Cosine similarity is relatively robust to outliers because it mainly measures the directional similarity between vectors rather than relying on their magnitude. As a result, the influence of extreme values is reduced, leading to more accurate similarity estimation in the presence of outliers.

The back propagation algorithm is used to update, and the target equation and the calculation process are as follows. First, centralize the data as follows:

(5) $$\bar{H_{1}^{\prime }}=H_{1}^{\prime }-\frac{1}{n}H_{1}^{\prime }$$

(6) $$\bar{H_{2}^{\prime }}=H_{2}^{\prime }-\frac{1}{n}H_{2}^{\prime }$$

where $\bar{H_{1}^{\prime }}=\left[h_{1}^{\left(1\right)},\ldots ,h_{1}^{\left(n\right)}\right]$, $\bar{H_{2}^{\prime }}=\left[h_{2}^{\left(1\right)},\ldots ,h_{2}^{\left(n\right)}\right]$. Data centering ensures that the mean of the centered sample is 0, facilitating data alignment and subsequent processing. For the projection vector pair $\left(h_{1}^{\left(i\right)},h_{2}^{\left(i\right)}\right)$ of the *i*-th sample, the cosine similarity is as follows:

(7) $$\cos\left(h_{1}^{\left(i\right)},h_{2}^{\left(i\right)}\right)=\frac{{\left(h_{1}^{\left(i\right)}\right)}^{T}h_{2}^{\left(i\right)}}{{\left\|h_{1}^{\left(i\right)}\right\|}_{2}{\left\|h_{2}^{\left(i\right)}\right\|}_{2}}$$

The optimization objective is defined as maximizing the mean cosine similarity among all pairs of samples, which serves as the objective function:

(8) $$\underset{\alpha_{1},\alpha_{2}}{\max}J=\frac{1}{n}\sum_{i=1}^{n}\frac{{\left(h_{1}^{\left(i\right)}\right)}^{T}h_{2}^{\left(i\right)}}{{\left\|h_{1}^{\left(i\right)}\right\|}_{2}{\left\|h_{2}^{\left(i\right)}\right\|}_{2}}=\mathrm{tr}\left(\frac{\bar{H_{1}^{\prime T}}\bar{H_{2}^{\prime }}}{\left|\bar{H_{1}^{\prime }}\right|\left|\bar{H_{2}^{\prime }}\right|}\right)=\cos\left(\bar{H_{1}^{\prime }},\bar{H_{2}^{\prime }}\right)$$

Here, tr(•) represents the trace of the matrix. $∥•{∥}_{2}$ represents the L2 norm of the vector set. The cosine similarity $\cos\left(\bar{H_{1}^{\prime }},\bar{H_{2}^{\prime }}\right)$ is calculated using the Frobenius norm of the vector set, and its gradient expression with respect to the sum of eigenvectors is as follows:

(9) $$\frac{\partial \cos\left(\bar{H_{1}^{\prime }},\bar{H_{2}^{\prime }}\right)}{\partial \bar{H_{1}^{\prime }}}=\frac{1}{\left|\bar{H_{1}^{\prime }}\right|\left|\bar{H_{2}^{\prime }}\right|}\bar{H_{2}^{\prime }}$$

(10) $$\frac{\partial \cos\left(\bar{H_{1}^{\prime }},\bar{H_{2}^{\prime }}\right)}{\partial \bar{H_{2}^{\prime }}}=\frac{1}{\left|\bar{H_{1}^{\prime }}\right|\left|\bar{H_{2}^{\prime }}\right|}\bar{H_{1}^{\prime }}$$

After training and optimizing deep neural networks and through backpropagation of $f_{1}(X_{1};\alpha_{1})$ and $f_{2}(X_{2};\alpha_{2})$, an approximate optimal solution for the parameters of the deep neural networks is obtained.

#### 3.2.4. Loss Optimization Module

To enhance the effectiveness of features, this paper designs a dual-loss joint optimization function that improves the overall performance of the model and achieves synergistic optimization of feature representation and classification decision by adjusting the weights of different loss functions. The expression of the adopted loss function is as follows:

(11) $$L=\omega_{1}\cdot L_{CE}+\omega_{2}\cdot L_{DCCA}$$

(12) $$L_{CE}=\frac{1}{n}\sum_{i=1}^{n}L_{i}=-\frac{1}{n}\sum_{i=1}^{n}\sum_{c=1}^{M}y_{ic}\log(p_{ic})$$

(13) $$L_{DCCA}=\mathrm{tr}\left(\frac{\bar{H_{1}^{\prime T}}\bar{H_{2}^{\prime }}}{\left|\bar{H_{1}^{\prime }}\right|\left|\bar{H_{2}^{\prime }}\right|}\right)$$

Among these, $L_{CE}$ is the cross-entropy loss; $L_{DCCA}$ is the loss based on cosine similarity for deep canonical correlation analysis; $\omega_{1}$ and $\omega_{2}$ represent weights ($\omega_{1}+\omega_{2}=1$); n is the total number of samples; M is the total number of categories; $y_{ic}$ is the sign function, i.e., ($y_{ic}\in \{0,1\}$); the true category c of sample i is 1, otherwise c is 0; $p_{ic}$ is the probability that sample i belongs to category c; $\bar{H_{1}^{\prime }}$ is the feature obtained by the gaze data after passing through the feature extractor; and $\bar{H_{2}^{\prime }}$ is the feature obtained by the pupil data after passing through the feature extractor. During the experiment, we observed that the magnitude of $L_{CE}$ is approximately ten times that of $L_{DCCA}$. To balance the influence of these two components, we adjusted the weighting parameters through multiple experiments. The ratio $\omega_{1}$:$\omega_{2}$ = 1:9 (i.e., 0.1 and 0.9) was found to provide the most stable training process and the best performance. This setting helps compensate for the difference in scale between $L_{CE}$ and $L_{DCCA}$, ensuring that both terms contribute appropriately during optimization. Consequently, in the experiment, $\omega_{1}$ is set to 0.1 and $\omega_{2}$ is set to 0.9.

## 4. Experimental Results and Analysis

### 4.1. Experimental Procedure

#### 4.1.1. Experimental Design

The research objective of this experiment is to classify different types of emotions through the proposed algorithm model. Currently, there are two mainstream methods for emotion classification: one is to classify specific emotions; the other is based on the two-dimensional affective model of valence–arousal proposed by Russell for emotion classification. These emotions are highly systematically correlated through positive or negative correlations, where valence is used to describe the negative or positive degree of an event, and arousal is used to describe the degree of interest in the event. Russell’s model can represent the changes between different emotions and can also more clearly express certain emotions that are difficult to describe. In this experiment, a combined method of the two classification approaches is adopted based on the characteristics of different datasets for classification and, by comparing with existing baseline algorithms, the superiority of the proposed method is intended to be fully validated. The data splitting strategy plays an essential role in model evaluation, as it defines the division of the dataset into training and testing sets and directly influences the model’s performance. In this study, all experiments employ a subject-independent classification method for dataset partitioning.

Traditional machine learning and classic deep learning methods, including multiple approaches from both categories, are selected as comparison objects. Traditional machine learning methods include mainstream algorithms such as SVM, random forest, and decision trees, while deep learning includes models such as CNN and LSTM. All comparison models use completely consistent dataset partition ratios, evaluation metrics, and hyperparameter optimization processes to avoid result bias caused by experimental condition differences from the source.

#### 4.1.2. Experimental Environment

The experiment’s software and hardware configurations are as follows: The hardware includes an NVIDIA GeForce RTX 4060 GPU with 12 GB of video random access memory. The software uses Python 3.12.3 and relies on libraries including Pandas, PyTorch 2.2.1, NumPy, and Scikit-learn 1.2.1.

#### 4.1.3. Experiment Parameter Configuration

All models in the experiment were trained using the same parameter inputs, and the input features of all models remained consistent. The Adam optimizer was used for training. The specific parameter configuration is shown in Table 2.

#### 4.1.4. Experiment Dataset

##### SEED-V Dataset

The SEED-V dataset [40] comprises five emotional categories: happy, sad, fear, disgust, and neutral. Sixteen healthy participants (6 males, 10 females) aged 19–28 were recruited. For each emotion, nine movie clips were selected based on their elicited emotional effects, with durations ranging from 2 to 4 min. The subjects in each experiment needed to watch 15 pieces of stimulating material and 3 pieces of each type of emotion. To study the stability of emotion recognition and ensure the effectiveness of the stimulation, each subject was asked to participate in the experiment three times, with at least three days between experiments.

In the self-assessment section, the subjects were asked to score according to the induction effect of the stimulus material. The scoring range was 0–5 points, of which 5 points represented the best induction effect and 0 points the worst. If the participant feels joy after watching the joy video, they should be scored 4–5 points, and if they do not feel anything, they should be given a score of 0. It is worthwhile to mention that if you are watching neutral stimulus material, if the subject’s mood fluctuates, the score should be 0, and the natural state is 5 points.

##### SEED-IV Dataset

The SEED-IV dataset [35] is similar to the SEED-V dataset in that it contains EEG and eye-movement signals recorded by 15 participants who watched a 2 min video clip to elicit four emotions: happy, sad, fear, and neutral. Each participant completed 6 trials for each emotion, resulting in 24 composite trials. The signals were down sampled to 200 Hz and segmented into non-overlapping 4 s intervals. Eye-movement signals were recorded using the SMI eye-tracking glasses.

##### eSEE-d Dataset

The eSEE-d dataset [26] is designed for the assessment of emotional states through eye-tracking data. Eye-movements of 48 participants were recorded while they viewed 10 emotionally evocative videos, each followed by a neutral clip. The collected signals encompassed various indicators of fixation, blinking, and pupil dynamics, allowing for the extraction of a comprehensive set of eye-tracking features, including fixations and saccades. Participants rated five emotions—tenderness, anger, disgust, sadness and neutral—on a scale from 0 to 10, which were subsequently transformed into emotional arousal and valence measures. Additionally, each participant completed three self-assessment questionnaires. A comprehensive analysis was conducted on the participants’ self-assessment scores on the questionnaires and their scores during the experiment. In this dataset, our study used the Russell model of the emotion coordinate system to convert the five emotions—anger, sadness, disgust, tenderness, and neutral—in the experiment into three categories of valence: positive valence (PV), medium valence (MV), and negative valence (NV); and three categories of arousal: high arousal (HA), medium arousal (MA), and low arousal (LA). Specifically: anger/disgust → high arousal—negative value (HANV), sadness → low arousal—negative value (LANV), tenderness → low arousal—positive value (LAPV), and neutral → medium arousal—medium value (MAMV). The statistical results after feature extraction are as follows: Classified by arousal, there were 1892 high arousal samples, 1605 low arousal samples, and 492 medium arousal samples.

#### 4.1.5. Data Preprocessing and Feature Extraction

As the experimental data in this study came from three publicly available datasets containing complete raw data, some of the raw data might become unusable due to unexpected or unforeseen errors during the experiment; consequently, it was necessary to convert these raw signals into structured inputs suitable for the research. During the experiment, we performed data preprocessing and feature engineering on the data.

The SEED-IV and SEED-V datasets already have fully classified eye-tracking features. In order to compare with other methods that also use the same datasets, this paper has not changed the features in the datasets. The eye-movement features are based on the SEED-IV and SEED-V datasets and are shown in Table 3.

The eSEE-d dataset comprises raw eye-tracking data, which necessitates specific pre-processing steps such as filtering, data alignment, and interpolation-based smoothing. Feature extraction is conducted on the unprocessed data and computing metrics include the mean and standard deviation of pupil diameter, fixation duration, saccade duration, as well as the kurtosis and skewness of saccade velocity. All proposed features are derived directly from the raw dataset.

To ensure the statistical significance of the extracted features, a significance test was conducted. Specifically, one-way analysis of variance (ANOVA) was employed to evaluate whether the extracted features exhibit statistically significant differences across different emotion categories. Features with *p*-values lower than 0.05 were retained, indicating that they contribute significantly to distinguishing emotional states. This procedure helps eliminate redundant or non-informative features and improves the efficiency and generalization capability of the proposed model. The eye-tracking features derived from the eSEE-d dataset are summarized in Table 4.

### 4.2. Experimental Results and Comparison

To demonstrate the superiority of this method, the proposed ICTD is compared with other methods in some literature. The goal is to verify the effectiveness of the proposed model. For the evaluation metric, we mainly use accuracy for comparison. The calculation formula is as follows:

(14) $$\mathrm{Accuracy}=\frac{TP+TN}{TP+FN+TN+FP}$$

In this context, true positive (*TP*) and false negative (*FN*) represent whether the target sample is classified correctly or incorrectly, while true negative (*TN*) and false positive (*FP*) represent whether the non-target sample is classified correctly or incorrectly.

#### 4.2.1. Evaluation of ICTD’s Effectiveness on the eSEE-d Dataset

In the experiments on the eSEE-d dataset, this paper classifies the data separately according to the specific emotions, and 5-fold cross-validation is conducted in the experiments. In the experiment, 80% of the data were selected as the training set, and the remaining 20% were used as the test set. Table 5 shows the classification results on the eSEE-d dataset and the comparison results with existing methods.

The experimental results show that the multimodal improved deep canonical correlation CNN–transformer method proposed in this paper has 81.8% arousal accuracy and 85.2% valence accuracy on this dataset. The 95% arousal confidence interval is [80.9%, 82.7%] and valence confidence interval is [84.2%, 86.2%], indicating excellent performance. Compared with traditional machine learning, this method’s recognition accuracy exceeds SVM’s highest respective arousal and valence accuracies of 64.6% and 70.1%, and the accuracy is improved by a respective 17.2% and 15.1%. Compared with the deep learning method, the accuracy of arousal and valence is improved by 10.5% and 12%, compared with the popular CNN–transformer method. Compared with the deep typical correlation analysis commonly used in the field of emotion analysis in recent years, the accuracy is improved by 6.3% and 8.9%, respectively. Compared with the literature that proposed the eSEE-d dataset, the accuracy of arousal and valence was improved by 3.0% and 1.2%, respectively.

Overall, the results show that our model has a better effect on eye-movement emotion classification, and the accuracy is greatly improved compared with traditional machine learning methods, indicating that it can achieve high accuracy even with a small input sample size. Compared with the current commonly used deep learning methods, it can also achieve better classification results.

**Figure 2:**
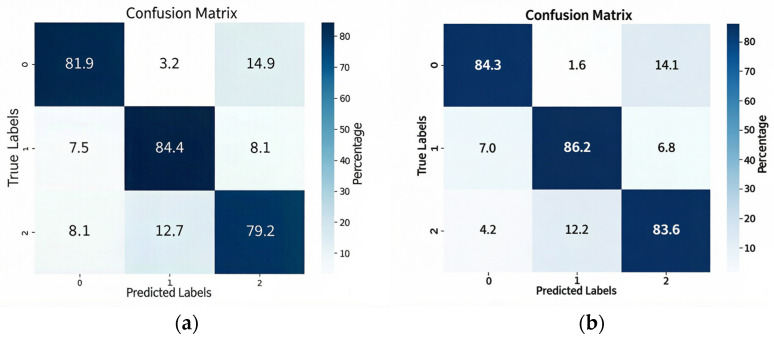
(**a**) Confusion matrix of accuracy for three-category arousal classification and (**b**) confusion matrix of accuracy for three-category valence classification.

To provide a more detailed analysis of the proposed method’s performance on this dataset, Figure 2 presents the standardized confusion matrices obtained by aggregating the results from all cross-validation folds. Here, Figure 2a shows a confusion matrix of accuracy for a three-category arousal classification, with the corresponding arousal categories being 0-LA, 1-MA, and 2-HA. Figure 2b shows a confusion matrix of accuracy for a three-category valence classification, with the corresponding arousal categories being 0-NV, 1-MV, and 2-PV. The values on the diagonal of the confusion matrix represent the accuracy of each emotion when using a single modality. The confusion matrix results show that the model’s accuracy in identifying valence is higher than its accuracy in identifying arousal. This is because the dataset uses five emotional stimuli to stimulate the subjects. Among these five emotions, the ratio of positive valence, medium valence, and negative valence is 1:1:3, while the ratio of high arousal, medium arousal, and low arousal is 2:1:2. Positive valence and medium valence each correspond to only one emotion, resulting in higher accuracy after the model classifies emotions. Arousal, on the other hand, has a more even distribution and is more difficult to distinguish than valence, hence the above results. This indicates that the model’s results conform to the objective laws of the data. Furthermore, the results show that the model performs best in both arousal and valence classification, reaching 84.4% and 86.2% for medium arousal and neutral valence, respectively. The main confusion in arousal occurs when 14.9% of “low arousal” is incorrectly classified as “high arousal,” while 14.1% of negative valence is classified as positive valence. This also proves that eye-tracking parameters differ significantly from other categories in medium valence and medium arousal, resulting in the best classification results.

#### 4.2.2. Evaluation of ICTD’s Effectiveness on the SEED-IV and SEED-V Datasets

As both SEED-IV and SEED-V datasets were proposed by Lu Baoliang’s team at Shanghai Jiao Tong University, and the experimental methods for both datasets are identical (differences only lie in the number of participants and the number of emotion categories), presenting the experimental results for both datasets simultaneously allows for a better comparison of the performance of the proposed method on both datasets. In the SEED-IV and SEED-V dataset experiments, this paper classifies the data separately according to the arousal and valence dimensions, and 5-fold cross-validation is conducted in the experiments. In the experiment, 80% of the data were selected as the training set, and the remaining 20% were used as the test set. Table 6 and Table 7 shows the classification results on the SEED-IV and SEED-V datasets and the comparison results with existing methods. Both datasets are multimodal datasets composed of EEG and eye-tracking data, only the eye-tracking data were used in this study.

Experimental results demonstrate that the proposed multimodal improved deep canonical correlation CNN–transformer method achieves accuracies of 91.2% and 85.1% on the SEED-IV and SEED-V datasets, respectively, and that the 95% confidence intervals are [89.1%, 93.3%] and [82.3%, 87.9%], exhibiting excellent performance. Compared with traditional machine learning, its recognition accuracy surpasses the highest arousal and valence accuracy of SVM by 72.3% and 68.6%, respectively, representing improvements of 19.9% and 16.5%. Compared with deep learning methods, and specifically the currently popular CNN–transformer method, it improves accuracy by 10.1% and 7.3% in arousal and valence, respectively. Compared with deep canonical correlation analysis, which has been commonly used in emotion analysis in recent years, it improves accuracy by 2.3% and 3.1%.

**Figure 3:**
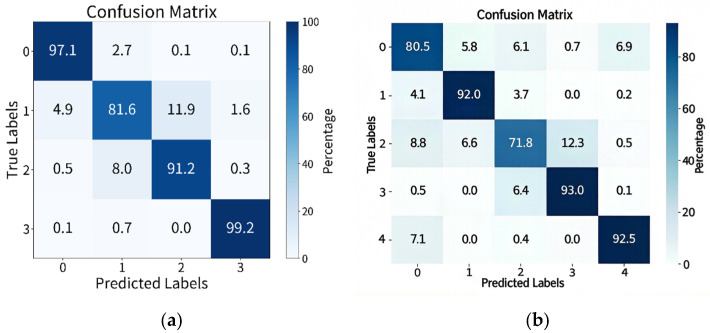
(**a**) Emotion classification accuracy confusion matrix of SEED-IV and (**b**) emotion classification accuracy confusion matrix of SEED-IV.

Figure 3 presents the confusion matrices illustrating the emotion classification performance on the SEED-IV and SEED-V datasets. Specifically, Figure 3a shows the confusion matrix for the SEED-IV dataset, where the emotion categories are defined as 0—neutral, 1—sad, 2—fear, and 3—happy. Figure 3b shows the confusion matrix for the SEED-V dataset, with the corresponding emotion categories being 0—disgust, 1—fear, 2—sad, 3—neutral, and 4—happy. Overall, the results show that our model outperforms both traditional machine learning methods and several commonly used deep learning methods listed. The experimental results on the SEED-V dataset are generally lower than those on SEED-IV because SEED-IV is a four-class emotion dataset, while SEED-V is a five-class emotion dataset, making classification more challenging.

#### 4.2.3. Ablation Experiment

To verify the functional contribution of each module in ICTD, this section conducts ablation experiments on the CNN–transformer module, IFFN module, and cos-DCCA module. To align the ablation results more closely with the model’s performance in practical applications, the ablation dataset is used for four-classification training based on the SEED-IV subject-independent experiment, with 5-fold cross-validation implemented. The results of the ablation experiments are summarized in Table 8. The results show that removing the IFFN module causes a 1.8% decrease in model accuracy, which means that replacing the FFN module in the transformer with IFFN can enhance the performance of the model. After removing the cos-DCCA module, the model accuracy drops by 5.7% with the largest accuracy deviation, which means the DCCA module founded on cosine similarity has the greatest impact on classification accuracy. In addition, in the experiment where the cos-DCCA module and IFFN module are removed, the model accuracy decreases by 10.1%, which means that using only the CNN–transformer method results in a significant deviation from the ICTD model’s outcomes.

## 5. Discussion

In order to solve the problems mentioned in Section 2, this paper proposes an improved CNN–transformer combined with enhanced deep canonical correlation analysis network, which mainly contributes as follows:

(1) Traditional eye-tracking emotion analysis mostly uses feature splicing or double-branch alignment to process data, with which it is difficult to capture the deep association and hierarchical structure between eye-movement features, in turn often leading to fragmentation of emotion representation. In this paper, CNN–transformer is combined with an enhanced deep canonical correlation analysis model. After using CNN and transformer to capture local and global information, it learns high-order nonlinear features, and then effectively integrates eye-movement features, explores the correlation between features, and improves the practicability of eye-movement features.

(2) Existing studies mostly rely on the statistical characteristics of the original data for emotion analysis. Moreover, the importance of each emotional feature in the calculation process is not primary or secondary, and the role of key features cannot be highlighted. To solve this problem, an incremental feature feedforward network module is designed to improve the transformer module. Replacing features from indiscriminate inputs by adding weight to key features increases the importance of these key features. This design effectively addresses the lack of prioritization in feature importance, and significantly improves the ability of eye-movement features to characterize emotional states.

However, the ICTD has certain limitations. While the model performs well on the same dataset its performance may vary across different subjects, experimental settings, or eye-tracking devices, indicating a need for improved cross-subject generalization. In addition, the IFFN architecture, combined with transformer and local convolution operations, increases both training and inference time, potentially restricting real-time emotion analysis applications.

## 6. Conclusions

This study proposes an improved CNN–transformer framework integrated with an enhanced deep canonical correlation analysis network for emotion classification based on eye-movement data. The proposed method addresses the limitation that a small number of eye-tracking features cannot adequately represent complex emotional states. In the proposed architecture, convolutional neural networks (CNNs) and transformer modules are employed to capture local and global feature representations, respectively. Furthermore, the transformer is enhanced by introducing an incremental feature feedforward network, which enables the model to assign greater importance to salient feature information. The extracted features are subsequently processed using deep canonical correlation analysis based on cosine similarity so as to obtain the final classification results. Experimental evaluations conducted on the eSEE-d, SEED-IV, and SEED-V datasets demonstrate the effectiveness of the proposed approach.

In future work, we plan to incorporate additional datasets to further expand the data scale. Moreover, deep multimodal fusion strategies combining eye-tracking features with other physiological signals will be explored to identify features that better reflect emotional states, thereby improving the robustness, practicality, and application potential of the proposed method.

## Figures and Tables

**Table 1:** Summary of methods and features in emotion classification.

Authors	Objectives	Modalities	Features	Results
Skaramagkas [26]	To test new machine learning methods and attempt to develop the best model that uses eye-tracking functionality to predict emotional states.	Eye	Fixation and blink frequency, saccade amplitude and duration, pupil diameter, fixation duration kurtosis, saccade duration variation and pupil diameter kurtosis.	84%
Fu [27]	To enhance the accuracy and stability of emotion recognition, a dual-branch feature extraction module is designed to extract features from both modalities simultaneously while maintaining temporal alignment between the modal signals.	EEG, Eye	33-dimension eye-movement features such as pupil diameter, gaze details, saccade details, blink details, and event details statistics, and a 62 channel EEG signal.	87%
Oliva [21]	Investigate the relationship between pupil size fluctuations and the process of emotion recognition. Participants heard human nonverbal vocalizations and indicated the emotional state of the speakers as soon as they had identified it.	Eye	Peak pupil dilation, the time of peak dilation, the rate of pre-peak dilation, and the rate of post-peak contraction.	81%
Li [28]	Develop a deep gradient convolutional neural network (DGCNN) using eye-movement signals for emotion classification.	Eye	Image features	87%
Aracena [23]	Using the evolution of the eye-tracking data during a window of time for recognizing the emotions which are provoked by visual stimuli of colored images labeled as negative, neutral and positive.	Eye	Pupil size average, pupil size change average	72%
Gong [31]	To overcome the individual differences caused by the non-stationary and low signal-to-noise properties that bring several challenges to cross-subject emotion recognition tasks, a novel multimodal emotion recognition model was proposed for improving the generalization performance to unseen target domain subjects.	EEG, Eye	33-dimension eye-movement features such as pupil diameter, gaze details, saccade details, blink details, and event details statistics, and a 62 channel EEG signal.	96%
Liu [33]	Identify the similarities and differences among Chinese, German, and French individuals in emotion recognition with electroencephalogram (EEG) and eye-movements from an affective computing perspective.	EEG, Eye	33-dimension eye-movement features such as pupil diameter, gaze details, saccade details, blink details, and event details statistics, and a 62 channel EEG signal.	84%
Tarnowski [29]	To classify three emotional states, the study employed support vector machines, linear discriminant analysis, and k-nearest neighbors, with performance evaluated using the leave-one-subject-out cross-validation approach.	Eye	18-dimension eye-movement features such as average duration of fixation, skewness of fixation durations, average amplitude of the saccades, variation of saccade amplitudes, average pupil diameter, pupil diameter variance, pupil diameter kurtosis.	80%

**Table 2:** Experimental parameter Configuration Table.

Category/Parameter	Value
Training and optimization	
Optimizer	Adam
Learning rate	0.0005
Momentum	(0.9, 0.999)
Batch size	128
Epochs	100 (eSEE-d), 50 (SEED-IV, SEED-V)
Decay rate	0.1
Weight decay	0.01
Model architecture	
Hidden layer1	64
Hidden layer2	128
Dropout	0.3

**Table 3:** Eye-tracking features extracted from the SEED-IV and SEED-V datasets.

Eye-Movement Parameters	Extracted Features
Pupil diameters (left and right)	Mean, standard deviation
Fixation duration (ms)	
Dispersion (X and Y)	
Saccade duration (ms)	
Saccade amplitude (°)	
Blink duration (ms)	
Event statistics	Fixation frequency, maximum fixation duration, maximum fixation dispersion, total fixation dispersion, saccade frequency, average saccade duration, average saccade amplitude, average saccade latency

**Table 4:** Eye-tracking features extracted from the eSEE-d dataset.

Eye-Movement Parameters	Extracted Features
Pupil diameter	Mean, Var, CV
Fixation duration (ms)	Mean, Var, CV
Saccade duration (ms)	Kurt, Skew, CV
Saccade speed	Kurt, Skew
Saccade distance	Kurt, Skew

**Table 5:** Classification accuracy comparison of various methods on the eSEE-d dataset.

Method	Arousal		Valence	
	Accuracy (%) ± Std	F1-Score (%) ± Std	Accuracy (%) ± Std	F1-Score (%) ± Std
SVM	64.6 ± 1.6	63.5 ± 1.5	70.1 ± 1.1	68.3 ± 1.2
Random forest	62.9 ± 1.3	61.8 ± 1.2	56.5 ± 0.8	54.8 ± 0.8
CNN	69.5 ± 2.2	68.2 ± 2.0	71.2 ± 2.6	70.1 ± 2.5
CNN–transformer	71.3 ± 1.8	70.0 ± 1.7	73.2 ± 2.3	73.5 ± 2.1
LSTM	71.8 ± 2.5	70.5 ± 2.3	72.6 ± 2.4	75.6 ± 2.4
DCCA [39]	75.5 ± 3.6	74.2 ± 3.3	76.3 ± 3.1	76.8 ± 3.0
DMLP [26]	72.0 ± \	\	84.0 ± \	\
DGCNN [28]	78.8 ± 1.5	77.5 ± 1.3	82.1 ± 1.3	81.5 ± 1.2
ICTD (ours)	81.8 ± 0.9	80.4 ± 0.7	85.2 ± 1.0	84.2 ± 0.9

**Table 6:** Accuracy comparison of various methods on the SEED-IV datasets.

Method	SEED-IV	
	Accuracy (%) ± Std	F1-Score (%) ± Std
SVM	72.3 ± 1.8	68.5 ± 2.1
Random forest	66.2 ± 1.5	63.1 ± 1.8
CNN	75.4 ± 2.9	71.7 ± 2.5
CNN–transformer	81.1 ± 2.6	78.4 ± 2.2
LSTM	80.3 ± 1.7	77.6 ± 1.4
DGCNN [28]	87.8 ± 1.6	84.5 ± 1.7
DCCA [39]	88.9 ± 2.8	85.7 ± 2.4
ICTD (Ours)	91.2 ± 2.1	89.3 ± 2.0

**Table 7:** Accuracy comparison of various methods on the SEED-V datasets.

Method	SEED-V	
	Accuracy (%) ± Std	F1-Score (%) ± Std
SVM	68.6 ± 2.3	67.5 ± 2.5
Random forest	59.1 ± 1.7	58.2 ± 1.9
CNN	71.2 ± 3.1	70.3 ± 3.3
CNN–transformer	77.8 ± 3.4	76.8 ± 3.6
LSTM	76.6 ± 1.6	76.0 ± 1.5
DGCNN [28]	82.1 ± 2.3	81.5 ± 2.5
DCCA [39]	82.0 ± 3.2	80.3 ± 3.4
ICTD (Ours)	85.1 ± 2.8	84.5 ± 3.0

**Table 8:** Results of ablation experiments.

Method	Accuracy (%) ± Std	F1-Score (%) ± Std
ICTD (ours)	91.2 ± 2.1	89.3 ± 2.0
w/o IFFN	89.4 ± 1.8	88.1 ± 1.7
w/o cos-DCCA	85.5 ± 1.3	84.9 ± 1.2
w/o IFFN + cos-DCCA	81.1 ± 2.6	78.4 ± 2.2

## Data Availability

The data presented in this study are openly available in reference number [eSEE-d] [https://doi.org/10.3390/brainsci13040589] [26]; [SEED-IV] [https://doi.org/10.1109/TCYB.2018.2797176] [35]; [SEED-V] [https://doi.org/10.1109/NER.2019.8716943] [40].

## Abbreviations

The following abbreviations are used in this manuscript:

IFFN	Incremental feature feedforward network
LA	Low arousal
MA	Medium arousal
HA	High arousal
NV	Negative valence
MV	Medium valence
PV	Positive valence
LSTM	Long short-term memory
KNN	k-nearest neighbors
SVM	Support vector machine
RNN	Recurrent neural network
DGCNN	Deep gradient convolutional neural network
DMLP	Deep multi-layer perceptron
STFT	Short-time Fourier transform
DCCA	Deep canonical correlation analysis
ANOVA	Analysis of variance
ICTD	Improved CNN-transformer combined with enhanced deep canonical correlation Analysis network
SEED	The SJTU emotion EEG dataset
SEED-FRA	SEED includes data of French subjects
SEED-VI	SEED includes 4 categories of emotional classification
SEED-V	SEED includes 5 categories of emotional classification
fNIRS	Functional near-infrared spectroscopy
